# 

**Published:** 1892-02

**Authors:** 


### CONTENTS—FEBRUARY, 1892.—VOL. VII, No. 8.

ORIGINAL COMMUNICATIONS—
Thephining for Cerebral Hemorrhage. By E. Lanphear, M. D ........ 275
Distension of Gall Bladder: Operation. By J. Cummings, M D ........ 278
Influenza: Ætiology, Symptoms and Treatment. By B. J. Nowierski, M. D ........ 280
Treatment of Trachoma by Expression. By. W. H. Baldinger, M. D ........ 283
CORRESPONDENCE—
Cancer: What it it? Causes and Treatment. By C. S. Reeves, M. D. ........ 284
SOCIETY NOTES—
Rockwall County Medical Society ........ 285
Pan-American Medical Congress Executive Committee 285
" " " in Columbia ........ 286
Central Texas Medical Society: Programme ........ 287
EDITORIAL—
The Prescription Druggist an Anachronism ........ 289
Absurd and Dangerous Prescriptions ........ 292
MEDICAL NEWS AND MISCELLANY ........ 293
BOOK NOTICES ........ 304
PUBLISHERS' NOTES ........ 306

